# The Impact of Maternal Phubbing on Toddlers’ Language Development and Subsequent Social Development: A Three-Month Time-Lagged Analysis

**DOI:** 10.3390/bs16040596

**Published:** 2026-04-17

**Authors:** Hyojin Ji, Taekmin Lee, Yujin Jang

**Affiliations:** Early Childhood Education, Gachon University, Seongnam 13120, Republic of Korea; kkuljin@hanmail.net (H.J.); leetaek@gachon.ac.kr (T.L.)

**Keywords:** maternal phubbing, toddler language development, toddler social development, short-term longitudinal design (time-lagged design)

## Abstract

This study investigates the structural relationships between maternal phubbing, toddlers’ language development, and their social development over a three-month interval. The sample consisted of 239 toddlers (aged 16–36 months) residing in Seoul and Gyeonggi-do, along with their mothers and classroom teachers. Data collection was conducted in two phases: the primary survey in May 2025 assessed maternal phubbing and toddlers’ language development through maternal reports, followed by a secondary survey in August 2025, where classroom teachers evaluated the toddlers’ social development. The findings revealed that while maternal phubbing did not significantly predict toddlers’ language development, it exerted a significant negative impact on their social development three months later. Additionally, toddlers’ language development was found to positively influence their subsequent social development. These results suggest that maternal phubbing and toddlers’ language development operate through independent pathways to influence social outcomes. By employing a three-month short-term longitudinal design (time-lagged design), this study identifies a direct path from maternal phubbing to social development and reaffirms the critical role of early language skills in fostering social competence during toddlerhood.

## 1. Introduction

Although infancy is a very brief period in human life, it is a critical period during which the foundations of human development are established. Every developmental foundation formed during this period serves as a core basis for subsequent growth. Since it is particularly important to build trust in the world and in humanity during infancy, primary caregivers—who exert the most direct influence on infants—must play a role in supporting infants to trust others and engage in diverse social experiences (Bowlby, 1982; Erikson, 1975).

In modern society, smartphones have moved beyond being simple communication tools to becoming essential devices in the lives of modern people (Gonçalves et al., 2020), and their usage rates continue to increase. According to the ‘2023 Children’s Media Usage Survey’ by the Korea Press Foundation (2023), the age of exposure to smart media has lowered even into infancy. According to the ‘2024 Survey on Smartphone Overdependence’ by the Korean Ministry of Science and ICT (2025), the proportion of the risk group for overdependence among toddlers and children showed the steepest upward trend across all age groups. As the age at which infants and young children encounter smart devices becomes increasingly younger and the risk of overdependence rises, there is growing interest in the impact of smartphone use on infants and young children (McDaniel, 2019; Wolfers, 2021).

Smartphones function as portable computers and possess the characteristics of diverse media. Mobile phones serve not only as communication tools but also as media for social network services (SNS), a means of satisfying recreational needs for hobbies or leisure activities, and as independent personal media that function as individual spaces for storing music, photos, and videos (Jang & Hong, 2019). As smartphones are used for such diverse purposes, usage time is gradually lengthening, and a significant number of users even experience conditions close to smartphone addiction. As the time individuals immerse themselves in smartphones increases, smartphones can ironically become tools that hinder face-to-face communication and interaction, contrary to their original developmental intent (Karadağ et al., 2015). Such concerns can be maximized during time spent between a person with a smartphone and a person without one, a representative example being the time spent between a parent using a smartphone and a young child who does not have one.

Due to the universalization of smartphones, the phenomenon of caregivers being immersed in smartphones during interactions with their children has become frequent. This has raised concerns that infants may be deprived of opportunities for interaction that they should imitate, which could negatively impact their language development. Among various factors hindering the development of infants and young children, one of the new social phenomena related to smartphone use is ‘phubbing.’ Phubbing is a neologism created by the Australian publisher Macquarie Dictionary, combining ‘phone,’ meaning a mobile phone, and ‘snubbing,’ meaning to ignore or treat coldly. Phubbing is a term that describes the act of ignoring someone in a social context by paying attention to a smartphone during an interaction with another person. Phubbing behavior not only has serious adverse effects on interpersonal relationships but can also cause various negative emotions in the other person and lead to conflict (Roberts & David, 2016). Although this term emerged relatively recently, it can be very easily found in the interpersonal relationships of modern people. In phubbing-related terminology, a ‘phubber’ refers to the actor who ignores the person sitting across from them by using a smartphone rather than interacting, while a ‘phubbee’ refers to the person who feels ignored because the other person is using a smartphone instead of interacting with them.

Mothers raising young children tend to easily immerse themselves in smartphones as a coping mechanism to relieve physical fatigue and social isolation resulting from parenting (McDaniel & Radesky, 2018). Furthermore, they are discussed as a group whose dependence on smart devices inevitably increases for the purpose of acquiring parenting information and communicating with the outside world (Radesky et al., 2016). Looking at studies in Korea, women in their 30s raising young children exhibit a strong desire for communication through messengers or SNS compared to other age groups, making them highly vulnerable to the risk of smartphone overdependence (Ministry of Science and ICT, 2024). Recent studies suggest that problematic social media use is not explained solely by frequency of use, but also by the psychological motives underlying such use (Lim, 2024; G.-H. Kim & Lim, 2025).

However, maternal phubbing is a behavior that can reduce responsiveness to an infant’s signals or needs. Maternal phubbing refers to a pattern of behavior where a mother’s attention is diverted by smartphone use during interactions with her child, resulting in reduced interaction or fragmented attention, comprising dimensions such as nomophobia, interpersonal conflict, self-isolation, and problem awareness (M. B. Kim et al., 2022; Roberts & David, 2016). Beyond observable interactional disruptions during mother–child interactions but also reflects broader patterns of problematic smartphone use, such as excessive dependence, interpersonal conflict, and social withdrawal. These underlying characteristics may reduce mothers’ attentional availability, thereby undermining the quality of mother–child interaction (McDaniel & Radesky, 2018).

Because the parent–child relationship during infancy and early childhood is a dependent relationship, there are reports (J. J. Lee, 2023) that it has a more serious impact than phubbing between adults. According to previous studies, maternal phubbing influences infants to lose trust in their mothers and feel alienated, and it adversely affects communication and relationship satisfaction (Wang et al., 2023). Consequently, maternal phubbing is predicted to ultimately have a negative impact on the formation of secure attachment (J. J. Lee, 2023) and to act as a negative factor in the development of infants, which requires face-to-face interaction and sensitive responsiveness (McDaniel & Radesky, 2018).

Infancy is the most critical period influenced by parents; most children acquire language through communication with their parents, and rich linguistic communication with the mother promotes language development in infancy. In particular, healthy language development during infancy has a lasting impact until after school age. According to previous studies, the more a mother speaks, the faster the infant’s vocabulary increases (Hoff & Naigles, 2002). Furthermore, when parents consistently respond to a child’s signals, the child cooperates and responds to the parents’ requests, and their cognitive and linguistic abilities improve through positive interactions with others (Davies, 2004). In other words, an infant’s language development is deeply intertwined with interaction with the mother.

Maternal phubbing fundamentally undermines the human-to-human interaction essential for language development by causing the mother to focus more on her smartphone than on the infant. Maternal phubbing deprives the infant of the opportunities for “scaffolding” emphasized by Vygotsky. As the mother’s gaze is diverted to the smartphone, non-verbal interactions decrease, failing to meet the child’s relational expectations. Smartphone-immersed mothers may struggle to accurately interpret the intentions behind their child’s behavior (Radesky et al., 2014), and eye contact, an important component of early caregiver–infant interaction related to attachment formation, may be disrupted (Raudaskoski et al., 2017). While the dimensions measured in this study—such as nomophobia and self-isolation—primarily reflect a mother’s psychological dependence on smartphones, they serve as proxies for reduced cognitive and emotional availability during interactions with their children. These underlying psychological traits increase the likelihood of attentional drifting, where a mother may be physically present but psychologically disengaged, thereby interfering with responsive caregiving (McDaniel & Radesky, 2018). Consequently, the observed effects in this study should be understood as the impact of problematic usage patterns that manifest as maternal phubbing in daily routines. In severe cases, this can lead to ignoring the child’s safety by failing to respond to their communication or emotional distress (Elias et al., 2021). Accordingly, maternal phubbing is predicted to have a negative impact on the child’s language development.

Toddlers exhibit a rapid increase in vocabulary beginning around 16 months, with a notable ‘vocabulary explosion’ typically occurring between 18 and 24 months (Fenson et al., 1994). As language development during this period is highly sensitive to caregiver interaction, toddlers under the age of three constitute a particularly vulnerable group for examining the effects of maternal phubbing (McGillion et al., 2024; Swanson, 2020). During this stage, interaction with the mother plays a pivotal role in supporting children’s language development and early social skills. However, maternal phubbing may disrupt these interactions by reducing maternal responsiveness and emotional engagement, consequently diminishing the quality of mother–child interaction (Carson & Kuzik, 2021).

The language and social development of infants are very closely related. Since language is not merely a means of communication but a core tool for cognitive development through interaction with others, delays in language development are intimately linked to delays in social development (Vygotsky, 1978). Tomasello (2017) argued that language and sociality cannot be separated because language is not simply a cognitive process of learning words, but stems from a fundamental social motivation to form relationships and share intentions with others. There are reports that children who showed language disorders in early childhood are more likely to experience a wide range of social development problems, such as social withdrawal, anxiety, and difficulties in peer relationships, compared to typical children when they reach adolescence (Beitchman et al., 2001). This trend has also been supported by studies showing that young children with lower receptive and expressive language abilities exhibit more socio-emotional and behavioral problems (Thurm et al., 2018). Ultimately, infants with superior language skills can clearly express their needs or emotions to others, which helps in building positive relationships with peers and adults. High-level language skills will contribute to increasing social competence by improving the ability to solve problems through the appropriate expression of one’s desires or feelings.

To date, most studies related to phubbing have emphasized the importance of maternal parenting attitudes and interaction (Hart & Risley, 1995) and have dealt with the negative impact of phubbing on children’s emotional and relational development (McDaniel & Radesky, 2018). However, considering that development in infancy progresses with various domains organically connected, research that integrally examines maternal phubbing, infant language development, and social development is currently insufficient. In particular, studies on language development in infancy are lacking compared to those on early childhood; given that language acquisition largely occurs in the early stages of life, research focused on infancy is even more important.

With this, previous studies have been limited to cross-sectional designs, which posed challenges in identifying the causal direction of the impact of maternal phubbing on infant development. Accordingly, this study aimed to confirm the effects of maternal phubbing through a predictive study utilizing a short-term longitudinal design. By examining maternal phubbing and infants’ language and social development at three-month intervals, this study intends to clarify developmental processes that were difficult to identify through existing cross-sectional research. The specific research questions of this study are as follows:

1. What are the trends in maternal phubbing, infant language development, and infant social development?
2. What are the structural relationships between maternal phubbing, infant language development, and infant social development?

## 2. Methods

### 2.1. Participants

In South Korea, childcare center enrollment rates for children aged 1 and 2 exceed 90% (Ministry of Education, 2025). The researcher initially contacted the directors of 31 childcare centers to explain the study’s purpose and request their cooperation. Through these centers, mothers of enrolled toddlers were invited to participate in an online survey, resulting in a total of 255 respondents. Approximately three months later, the classroom teachers of the corresponding toddlers assessed the children’s social development. Following the exclusion of cases with significant missing values or unmatched mother–teacher data, a final sample of 239 toddlers was included in the final analysis.

Regarding the mothers’ age, those in their 30s constituted the majority (n = 174, 72.8%), and employed respondents (n = 183, 76.6%) were more prevalent than those who were unemployed (n = 56, 23.4%). Households with one child accounted for over half of the sample (n = 122, 51.0%), and the most common educational attainment was a four-year university degree (n = 151, 63.2%). The prevalent average monthly household income fell between 4 million and 7.99 million KRW (n = 111, 46.4%), which corresponds to the third and fourth quintiles of income groups in Korea (National Data Portal, 2025). Finally, the most frequently reported daily smartphone usage time among mothers was “between 2 and 3 h” (n = 82, 34.3%).

The gender of the toddlers consisted of 128 females (53.6%) and 111 males (46.4%), and the majority were either first-born or only children (n = 153, 64.0%). Regarding the age in months, toddlers aged 24–36 months occupied a high proportion at 160 participants (66.9%), and the time toddlers spent at institutions was most frequently “at least 5 h but less than 7 h” (n = 124, 51.9%). For the toddlers’ own smartphone usage time, 134 (56.1%) responded “do not use,” and 75 (31.4%) responded “less than one hour.”

### 2.2. Measures

#### 2.2.1. Maternal Phubbing

To measure maternal phubbing, this study utilized the Korean Phubbing Scale (PS-K), which was adapted and validated into a Korean version by M. B. Kim et al. (2022) based on the scale developed by Chotpitayasunondh and Douglas (2018). The sub-factors of the scale consist of a total of 15 items: Nomophobia (4 items), which refers to feeling anxious and irritable in the absence of a smartphone; Interpersonal conflict (4 items), which involves recognizing conflicts with others that occur when using a smartphone; Self-isolation (4 items), which involves using a smartphone to isolate oneself from others to withdraw from social activities; and Problem awareness (3 items), where the phubber recognizes that they themselves have difficulties in smartphone use. It is a 5-point Likert scale, where higher scores indicate a higher degree of phubbing behavior. The overall Cronbach’s α value for the scale’s reliability was 0.84. In addition, these sub-dimensions represent patterns of problematic smartphone use that may function as underlying mechanisms of maternal phubbing, as they are associated with reduced attentional control and responsiveness in parent–child interactions (McDaniel & Radesky, 2018).

#### 2.2.2. Language Development

To measure the language ability of toddlers, the Sequenced Language Scale for Infants (SELSI), a standardized assessment tool developed by Y. T. Kim et al. (2003), was utilized. This scale is designed to evaluate the overall language ability of toddlers under the age of three, from 5 months to under 36 months, and the language development characteristics occurring at the respective months are divided by age. It consists of a total of 112 items, including 56 items for receptive language and 56 items for expressive language.

Based on maternal reports, a basal level is established when eight consecutive “yes” responses are given, and the response stops at the ceiling level when eight consecutive “no” responses occur. The respondent mother reads the items herself and answers “yes” or “no,” with the rating being “yes (1 point)” and “no (0 points).” The overall Cronbach’s α value for the scale’s reliability was 0.84, with receptive language at 0.96 and expressive language at 0.74.

#### 2.2.3. Social Development

To measure the social development of toddlers, this study used the scale of social-emotional development rating scale developed by H. I. Kim (2010) for Korean toddlers. In this study, 16 items for self-regulation and 9 items for social relationships were used. As a result of conducting an exploratory factor analysis for this study, items from the self-regulation sub-factor (Nos. 13, 14, 15, and 16) where factor loading values were below the threshold or clustering between factors was unclear were deleted. Consequently, a total of 21 items, consisting of 12 items for self-regulation and 9 items for social relationships, were used for the final analysis.

Although the original scale can be rated by either parents or teachers, data for this study were collected through teacher ratings. This was based on research (Jang, 2018) stating that information on toddlers’ social-emotional development is more accurate when reported by teachers, as teachers can compare and analyze more toddlers than parents. The overall Cronbach’s α value for the scale’s reliability was 0.88.

### 2.3. Data Analysis

The data analysis plan was established a priori. Data were analyzed using IBM SPSS Statistics 25 and AMOS 22. Descriptive statistics and frequency analyses were employed to examine the demographic characteristics of the participants, and Cronbach’s alpha was calculated to assess reliability. Pearson correlation analysis was conducted to assess the associations among variables.

A confirmatory factor analysis (CFA) was performed using AMOS 22 to evaluate the measurement model. Structural equation modeling (SEM) was then utilized to examine the mediating effect of toddlers’ language development on the link between maternal phubbing and toddlers’ social development. Toddler gender and average monthly household income were included as control variables. Finally, bootstrapping was conducted to test the significance of direct, indirect, and total effects, using 95% confidence intervals.

## 3. Results

### 3.1. Descriptive Statistics of Main Variables

The descriptive statistics for the major variables in this study are presented in Table 1. The overall mean for maternal phubbing was 2.21 (SD = 0.53), which is lower than the median. Among its sub-factors, interpersonal conflict and self-isolation showed very low scores, whereas nomophobia and problem awareness were at moderate levels. The overall mean for Language Development was 94.01 (SD = 17.50), which is higher than the median, with Receptive Language scores being slightly higher than Expressive Language scores. Notably, the standard deviation for Expressive Language (SD = 10.27) was larger than that for Receptive Language (SD = 8.08), indicating that the developmental gap among toddlers is more pronounced in language production skills. The overall mean for toddlers’ Social Development was 3.71 (SD = 0.53), a level higher than the median.

In addition, the normality of the data was assessed by examining skewness and kurtosis. All variables met the acceptable criteria for normality (absolute skewness < 2, absolute kurtosis < 7), indicating no substantial deviation from normality (West et al., 1995). Therefore, parametric statistical methods were deemed appropriate for subsequent analyses.

### 3.2. Correlations Among Main Variables

To identify the relationships between the major variables, Pearson’s correlation coefficient analysis was conducted, and the results are shown in Table 2. Among the sub-factors of Maternal Phubbing, Interpersonal Conflict showed a significant negative correlation (r = −0.16, *p* < 0.05) with Self-Regulation 2, a sub-factor of toddlers’ Social Development. Although it did not reach the statistical significance level of *p* < 0.05, Problem Awareness (r = −0.12) also approached statistical significance for a negative correlation with toddlers’ Self-Regulation at the 0.10 level. Surprisingly, Self-Isolation among the phubbing sub-factors showed a positive correlation with Expressive Language (r = 0.15, *p* < 0.05), which was contrary to expectations. The sub-factors of toddlers’ Language Development and Social Development showed a very high positive correlation.

### 3.3. Structural Equation Model Analysis

Prior to testing the structural model, confirmatory factor analysis was conducted to ensure the adequacy of the measurement model. All factor loadings were within acceptable ranges, supporting the validity of the measurement model. The model fit results are presented in Table 3. The χ^2^ was 54.93 and the χ^2^/df was 1.37, satisfying general criteria and indicating good model fit. The RMSEA was 0.04, NFI was 0.92, TLI was 0.97, and CFI was 0.98, with all indices exceeding the threshold values, demonstrating excellent fit.

**Figure 1:**
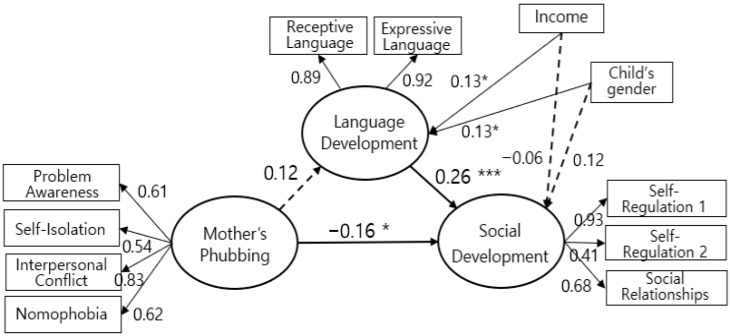
Final Model. * *p* < 0.05, *** *p* < 0.001.

This study conducted Structural Equation Modeling (SEM) to verify whether toddlers’ Language Development plays a mediating role in the effect of Maternal Phubbing on toddlers’ Social Development; the child’s gender and average monthly household income were included as control variables Figure 1, Table 4. Maternal Phubbing did not exert a direct effect on toddlers’ Language Development. However, toddlers’ Language Development had a significant direct effect (β = 0.26, *p* < 0.001) on their Social Development (comprising Self-Regulation 1, Self-Regulation 2, and Social Relationships) three months later. This implies that higher levels of language development lead to higher levels of social development in toddlers. Meanwhile, Maternal Phubbing had a significant direct negative (−) effect (β = −0.16, *p* < 0.05) on toddlers’ Social Development, illustrating the possibility that impaired interaction caused by maternal phubbing hinders social development. Among the control variables, the child’s gender (β = 0.13, *p* < 0.05) and monthly income (β = 0.13, *p* < 0.05) significantly influenced Language Development, whereas neither gender nor income significantly affected Social Development.

## 4. Discussion

The present study aimed to analyze the impact of maternal phubbing on toddlers’ Language Development and their Social Development three months later, and to examine the mediating role of Language Development in this relationship. Data were collected from 239 mothers of toddlers aged 16 to 36 months attending childcare centers in Seoul and Gyeonggi-do, as well as from their classroom teachers. The discussion based on the results is as follows.

First, maternal phubbing was found to be at a level lower than the median. This suggests that mothers reported relatively lower levels of smartphone use while spending time with their children. Among the sub-factors of phubbing, Nomophobia and Problem Awareness were relatively high, suggesting that mothers reported higher levels of anxiety related to smartphone absence and awareness of their smartphone use. Toddlers’ Language Development was at a favorable level, with Receptive Language scores higher than Expressive Language scores. This aligns with previous research (Fenson et al., 1994) suggesting that in the nature of infant and toddler language development, comprehension ability precedes production ability. Meanwhile, toddlers’ Social Development was generally positive, with both Self-Regulation and Social Relationships scoring above the median.

Second, Interpersonal Conflict, a sub-factor of maternal phubbing, showed a negative correlation with toddlers’ Self-Regulation. This implies that mothers’ perceptions of experiencing conflict due to smartphone use are related to the lower levels of self-regulation in their toddlers as rated by teachers. Mothers having Problem Awareness regarding their smartphone use also showed a marginal negative correlation with toddlers’ Self-Regulation. A very high positive correlation was confirmed between toddlers’ Language Development and Social Development. This result supports Vygotsky’s theory and prior research (Tomasello, 2017) that language is not merely a means of communication but a core tool for establishing relationships and demonstrating social competence. In other words, toddlers with superior language abilities are more likely to express their needs clearly and understand others well, leading to positive social relationships.

In particular, correlation analysis revealed a small but significant positive association between maternal phubbing and infants’ expressive language scores, and the magnitude of this association was similar to the negative correlation observed between phubbing and social relationships. However, this effect was not statistically significant in the structural equation model (SEM). This discrepancy may reflect differences between bivariate and multivariate analyses, suggesting that the observed association may be influenced by other variables rather than representing a direct effect. Therefore, this finding should be interpreted with caution. Furthermore, the finding that language development has a strong positive effect on social development suggests that the influence of maternal phubbing on language development may operate through indirect and complex pathways. Therefore, future research should more closely examine the relationship between expressive language and phubbing.

Third, maternal phubbing was found to exert a direct negative impact on toddlers’ Social Development three months later. This is consistent with previous findings that parental phubbing hinders social development by making children feel ignored, thereby undermining attachment security (J. J. Lee, 2023), weakening the parent–child bond (Pancani et al., 2021), and lowering the quality of interaction (Carson & Kuzik, 2021). However, in this study, the direct effect of maternal phubbing on toddlers’ Language Development was not significant. This somewhat contradicts studies reporting that maternal phubbing hinders toddlers’ language development (Song & Jang, 2021; Reed et al., 2017). On the other hand, it aligns with research suggesting that phubbing has a significant negative impact when mediated by the quality of the parent–child relationship rather than causing direct developmental impairment (David & Roberts, 2017), and that direct causal conclusions regarding the effect of parental smartphone use on children’s language development remain limited.

Speculating on the reasons why maternal phubbing did not significantly impact Language Development in this study, one might consider that most of the toddlers in this sample spend long hours in childcare centers. This suggests the possibility that by receiving sufficient linguistic stimulation from teachers and peers at the center, the linguistic deficit caused by maternal phubbing may be partially compensated. Despite the lack of an observed effect from phubbing on language, Language Development exerted a strong positive influence on Social Development three months later. These results support Sameroff’s (2010) integrative developmental model, suggesting that language ability is a key foundation for peer relationships and social competence (Beitchman et al., 2001) and that toddler development operates integrally rather than in isolation.

Based on these results, the following suggestions are drawn. First, it may be cautiously suggested that toddlers’ language development is influenced by multiple environments, including both the home and childcare settings, rather than relying solely on maternal factors. Furthermore, as the age of exposure to smart devices decreases, the link between home and childcare centers must be strengthened to support language and social development. Both environments should emphasize human-to-human interaction that digital devices cannot replace. Childcare centers should prioritize person-centered curricula and foster environments where humans interact and play face-to-face.

Second, specific parent education should be provided for parents of toddlers. Maternal phubbing was shown to have a direct negative impact on Social Development after three months, even without being mediated by language development. This implies that the lack of eye contact and delayed responsiveness occurring when a mother’s attention is captured by a smartphone may cause toddlers to lose the joy of interaction and diminish their ability to read social cues. Parent education should prioritize the importance of putting down smart devices and responding sensitively to a toddler’s non-verbal signals rather than simply talking to the child more. Recent intervention studies suggest that digital technology itself is not inherently harmful, as its effects depend largely on how it is used (Min et al., 2024; Shin et al., 2025). Additionally, programs could include practicing specific non-verbal interactions that are easily neglected due to phubbing. Parents should be informed that excessive media exposure at home can weaken a toddler’s language and social development, and efforts must be made to ensure that home and institutions provide a consistent caregiving environment. We must not forget the warning that many parents underestimate the risks of their children’s smart device use.

Moreover, since Language Development was a powerful predictor of Social Development, linguistic intervention could be an effective strategy for promoting social competence. Environments that help toddlers express their needs and emotions through appropriate language eventually lead to positive relationship-building with peers and adults. Institutions and homes should foster the foundations of social development through customized play programs that consider each toddler’s individual language level. Language education programs for improving social development could also be considered, as language is the basis of social competence for regulating one’s own needs and relating to others.

Third, future research should consider both direct and indirect exposure to smart media in toddlers. While this study focused on maternal phubbing, toddlers’ language development is likely influenced by a combination of parental interaction and their own experiences with digital media. In addition, the potential impact of other screen devices, such as tablets and televisions, during mother–child interactions should be examined (J. Lee, 2014). Moreover, because maternal phubbing was assessed when toddlers were between 16 and 36 months of age, the concurrent relationship with language development may not have been fully captured. Given that earlier developmental stages, particularly during the first and early second year of life, are highly sensitive to caregiver interaction (Tomasello, 2017), future research should examine the effects of phubbing at earlier ages.

Finally, this study is subject to potential same-reporter bias, stemming from the reliance on maternal self-reports to assess both maternal phubbing and toddlers’ language development. This approach may introduce common method bias, potentially inflating or attenuating the observed associations between variables. Furthermore, the measurement of maternal phubbing focused on self-reported psychological patterns rather than direct, real-time observation of smartphone use in the child’s presence. Although these sub-dimensions function as robust indicators of problematic use that likely disrupt maternal responsiveness, they do not fully capture the immediate, situational frequency of phubbing behaviors during specific interactional episodes. Consequently, the associations identified in this study should be interpreted with caution. Future research should address these limitations by incorporating multiple informants—such as spousal reports—or utilizing objective measures, including ecological momentary assessment (EMA) or video-coded behavioral observations, to more accurately capture the dynamic nature of maternal phubbing.

## 5. Conclusions

This study finds that while maternal phubbing among mothers of toddlers in Korean society remains at a moderate level, its direct negative impact on toddlers’ social development over a three-month period is statistically significant. The anticipated mediating role of language development was not observed—potentially due to the compensatory effects of high-quality childcare centers. What is especially noteworthy is that the relationship between maternal phubbing and language development appears to be more complex than its relationship with social development. A small but significant positive correlation was observed between maternal self-isolation and children’s expressive language. However, this association was not supported in the structural equation model, suggesting that it may not represent a direct effect. Therefore, this finding should be interpreted with caution, and it may reflect indirect or context-dependent relationships rather than a meaningful positive influence of maternal phubbing. The influence of language skills on subsequent social competence underscores the integrated nature of early development.

However, the influence of language skills on subsequent social competence underscores the integrated nature of early development. These findings emphasize that parental smartphone use does more than simply reduce verbal input; it fundamentally disrupts the delicate socio-emotional exchanges necessary for fostering self-regulation and interpersonal skills. Consequently, there is a need for parent education programs that move beyond screen-time management to prioritize sensitive, non-verbal responsiveness and the restoration of human-centered interactions within the home environment.

## Figures and Tables

**Table 1:** Descriptive Statistics of the Main Variables (N = 239).

		*M*	*SD*	*Min*	*Max*	Skewness	Kurtosis
Mother’s Phubbing	Nomophobia	2.76	0.73	1.00	4.50	−0.11	−0.44
	Interpersonal Conflict	1.68	0.58	1.00	3.75	0.66	0.06
	Self-Isolation	1.60	0.62	1.00	3.50	0.85	−0.24
	Problem Awareness	2.81	0.87	1.00	4.67	−0.40	−0.38
	Total	2.21	0.53	1.06	3.46	−0.02	−0.41
Language Development	Receptive Language	48.78	8.08	20.00	56.00	−1.23	0.78
	Expressive Language	45.23	10.27	17.00	56.00	−0.57	−0.89
	Total	94.01	17.50	37.00	112.00	−0.81	−0.31
Social Development	Self-Regulation1	3.81	0.64	2.00	5.00	−0.34	−0.16
	Self-Regulation2	3.31	0.78	1.00	5.00	−0.21	0.01
	Social Relationships	3.91	0.62	2.11	5.00	−0.17	−0.51
	Total	3.71	0.53	2.24	4.90	−0.10	−0.27

**Table 2:** Correlations among the Main Variables (*N* = 239).

	1	2	3	4	5	6	7	8	9
1	1								
2	0.52 ***	1							
3	0.23 ***	0.47 ***	1						
4	0.43 ***	0.47 ***	0.35 ***	1					
5	0.01	0.01	0.10	0.05	1				
6	0.02	0.08	0.15 *	0.06	0.82 ***	1			
7	−0.05	−0.02	0.06	−0.04	0.22 ***	0.25 ***	1		
8	−0.08	−0.16 *	−0.00	−0.12 ^†^	0.16 *	0.17 **	0.37 ***	1	
9	−0.04	0.06	0.03	−0.02	0.04	0.10	0.63 ***	0.31 ***	1

Note. 1. Nomophobia, 2. Interpersonal Conflict, 3. Self-Isolation, 4. Problem Awareness, 5. Receptive Language, 6. Expressive Language, 7. Self-Regulation 1, 8. Self-Regulation 2, 9. Social Relationships. ^†^ *p* < 0.10, * *p* < 0.05, ** *p* < 0.01, *** *p* < 0.001.

**Table 3:** Model Fit Indices.

	χ^2^	χ^2^/df	RMR	GFI	AGFI	NFI	IFI	TLI	CFI	RMSEA
Criteria				≥0.90	≥0.90	≥0.90	≥0.90	≥0.90	≥0.90	<0.08
Measurement Model	54.93	1.37	0.20	0.96	0.94	0.92	0.98	0.97	0.98	0.04

**Table 4:** Path Analysis and Mediation Analysis of the Research Model.

Path	Estimate		S.E.	t
	*B*	*β*		
Phubbing → Language Development	1.53	0.12	0.91	1.69
Phubbing → Social Development	−0.13	−0.16	0.07	−2.05 *
Language Development → Social Development	0.02	0.26	0.01	3.31 ***

* *p* < 0.05, *** *p* < 0.001.

## Data Availability

The datasets generated and/or analysed during the current study are not publicly available due to privacy and confidentiality restrictions.
